# Entanglement-Assisted Joint Monostatic-Bistatic Radars

**DOI:** 10.3390/e24060756

**Published:** 2022-05-26

**Authors:** Ivan B. Djordjevic

**Affiliations:** Department of Electrical and Computer Engineering, University of Arizona, 1230 E. Speedway Blvd., Tucson, AZ 85721, USA; ivan@email.arizona.edu; Tel.: +1-520-626-5119

**Keywords:** entanglement, radars, quantum sensing, quantum radars, entanglement assisted detection

## Abstract

With the help of entanglement, we can build quantum sensors with sensitivity better than that of classical sensors. In this paper we propose an entanglement assisted (EA) joint monostatic-bistatic quantum radar scheme, which significantly outperforms corresponding conventional radars. The proposed joint monostatic-bistatic quantum radar is composed of two radars, one having both wideband entangled source and EA detector, and the second one with only an EA detector. The optical phase conjugation (OPC) is applied on the transmitter side, while classical coherent detection schemes are applied in both receivers. The joint monostatic-bistatic integrated EA transmitter is proposed suitable for implementation in LiNbO_3_ technology. The detection probability of the proposed EA joint target detection scheme outperforms significantly corresponding classical, coherent states-based quantum detection, and EA monostatic detection schemes. The proposed EA joint target detection scheme is evaluated by modelling the direct radar return and forward scattering channels as both lossy and noisy Bosonic channels, and assuming that the distribution of entanglement over idler channels is not perfect.

## 1. Introduction

The entanglement represents a unique quantum information processing (QIP) attribute [1,2,3,4,5,6,7] that enables: (1) outperforming classical sensors sensitivity [1,2,5], (2) unconditional security for future communication networks [1,3,5,6], and (3) beating the classical channel capacities [8,9,10]. Further, the pre-shared entanglement enables distributed quantum sensing [1,7] and secure distributed quantum computing [11].

One of the key motivations behind the quantum target detection studies is to outperform the quantum limit of classical sensors [12]. The quantum radars have several advantages compared to corresponding classical counterparts: improved receiver sensitivity, better detection probability of targets, in particular in a low signal-to-noise ratio (SNR) regime, improved synthetic-aperture radar imaging quality, improved detection through clouds and fog (in particular when microwave photons are used), better resilience to jamming, and higher cross-section (as shown in [12]), to mention few. Moreover, the quantum radar signals are more difficult to detect compared to classical radars. On the other hand, the quantum radars are much more difficult to implement in practice. Recently, two popular quantum radar designs emerged: (i) the quantum radar employing Lloyd’s quantum illumination sensing concept [13] and (ii) interferometric quantum radar. For further details on various quantum radars concepts and classification of different quantum radar techniques an interested reader is referred to [14,15,16,17,18,19].

**Figure 1:**
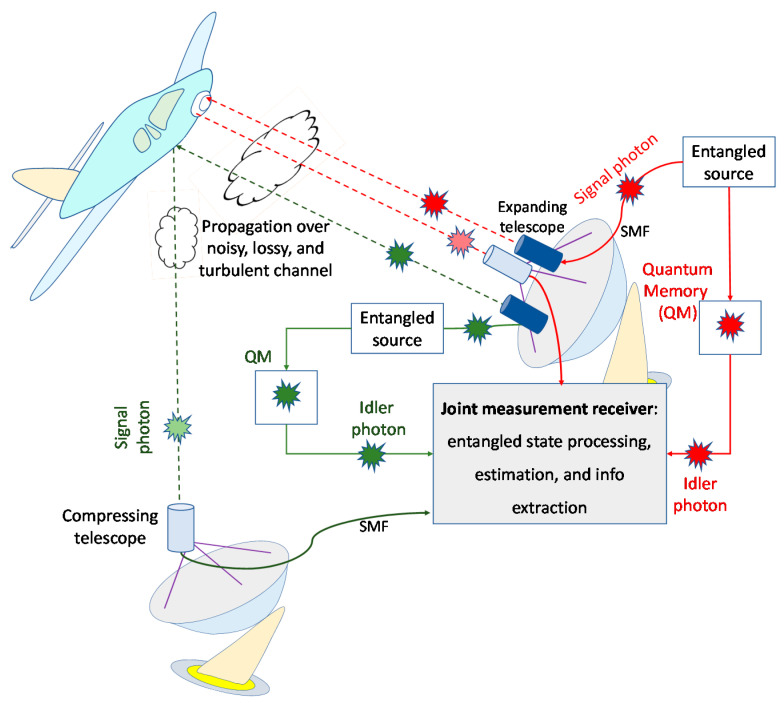
The proposed EA joint monostatic-bistatic quantum radar technique.

In this paper, we propose an entanglement assisted (EA) joint monostatic-bistatic quantum radar detection scheme with corresponding operational principle being depicted in Figure 1. The wideband entangled source generates two entangled pair of photons, each pair containing signal and idler photons. The idler photons are kept in the quantum memories of the receivers. Both signal photons are transmitted with the help of corresponding expanding telescopes over noisy, lossy, and atmospheric turbulent channel towards the target. Directly reflected photon is collected by the compressing telescope and detected by the first radar receiver, while the forward scattered photon is collected by the second compressing telescope and detected by the second radar receiver. The quantum correlation is utilized on receive sides to improve overall target detection probability. Inherent spatial diversity is exploited to improve the overall SNR. Additional description of the proposed joint monostatic-bistatic radar scheme is provided in Section 3.

To simplify design and at the same time improve the target detection probability we apply the optical phase conjugation (OPC) on the transmitter side rather than the receive side. We propose the joint monostatic-bistatic integrated EA transmitter that is suitable for implementation in LiNbO_3_ technology. The EA detectors are based on classical coherent detection with idler mode having the same role as the local oscillator (LO) laser signal. We show that the proposed EA joint target detection scheme significantly outperforms coherent states-based quantum detection, EA monostatic, and classical radar counterparts. We further evaluate the proposed EA joint target detection scheme by modelling both directly reflected mode channel and forward scattered mode channel as lossy and noisy Bosonic channels. Finally, we assume that the distribution of entanglement over the idler channels is not perfect.

The paper is organized as follows. The EA monostatic radar concept is introduced in Section 2, which is used as a reference case. The proposed EA joint monostatic-bistatic radar scheme, employing the OPC on the transmitter side and coherent detection on the receiver sides, is described in Section 3. Both directly reflected (return) signal mode and forward scattered signal mode channels are modeled as lossy and noisy Bosonic channels. The idler channels are also modelled as lossy and noisy Bosonic channels. In Section 4 we evaluate the detection probability performances of the proposed EA joint monostatic-bistatic radar target detection scheme and compare it against coherent states-based quantum detection, EA monostatic detection, and classical detection schemes. The relevant concluding remarks are given in the last section (Section 5).

## 2. Entanglement Assisted Monostatic Radars

**Figure 2:**
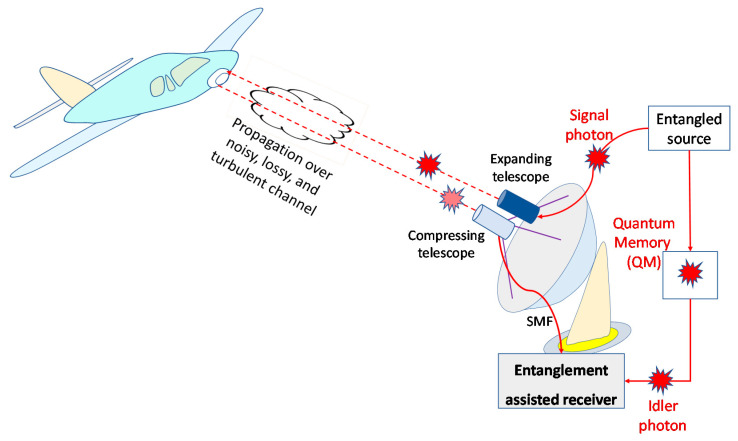
The EA monostatic quantum radar.

In this section, we describe the entanglement assisted monostatic radar target detection scheme, shown in Figure 2, employing the Gaussian states generated through the continuous-wave spontaneous parametric down conversion (SPDC) process. The SPDC-based entangled source represents a broadband source having *D* = *T_m_W* i.i.d. signal-idler photon pairs, where *T_m_* is the measurement interval and *W* is the phase-matching SPDC bandwidth. Each signal-idler photons pair, which for monostatic radar are denoted as red photons in Figure 2, is in fact a two-mode squeezed vacuum (TMSV) state whose representation in Fock basis is given by:

(1) $${|\psi \rangle }_{s,i}=\frac{1}{\sqrt{N_{s}+1}}\sum_{n=0}^{\infty }{\left(\frac{N_{s}}{N_{s}+1}\right)}^{n/2}{|n\rangle }_{s}{|n\rangle }_{i},$$

where $N_{s}=\langle {\hat{a}}_{s}^{†}{\hat{a}}_{s}\rangle =\langle {\hat{a}}_{i}^{†}{\hat{a}}_{i}\rangle$ is the mean photon number per mode, with corresponding signal and idler creation operators being denoted by ${\hat{a}}_{s}^{†}$ and ${\hat{a}}_{i}^{†}$, respectively. The signal-idler entanglement is characterized by the phase-sensitive cross-correlation (PSCC) coefficient, defined as $\langle {\hat{a}}_{s}{\hat{a}}_{i}\rangle =\sqrt{N_{s}(N_{s}+1)}$, which can be considered as the quantum limit.

The TMSV state represents a pure maximally entangled zero-mean Gaussian state with the following Wigner covariance matrix:

(2) $$\Sigma_{TMSV}=\left[\begin{array}{cc} \left(2N_{s}+1\right)1 & 2\sqrt{N_{s}\left(N_{s}+1\right)}Z\\ 2\sqrt{N_{s}\left(N_{s}+1\right)}Z & \left(2N_{s}^{}+1\right)1 \end{array}\right],$$

where ***Z*** = diag(1, −1) denotes the Pauli *Z*-matrix and **1** denotes the identity matrix. Clearly, in the low-brightness regime *N_s_* << 1, the PSCC is $\langle {\hat{a}}_{s}{\hat{a}}_{i}\rangle \approx \sqrt{N_{s}}$ that is much larger than the corresponding classical limit *N_s_*. As described earlier, by going back to Figure 2, the entangled source is used on the transmitter side to generate quantum correlated signal photon (probe) and idler photon, which serves as a local reference. With the help of the expanding telescope, the signal photon is transmitted over a noisy, lossy, and atmospheric turbulent channel towards the target. The reflected photon (the radar return) is collected by the compressing telescope and detected by the radar’s receiver, and the quantum correlation between radar return and retained reference (idler photon) is exploited on receive side to improve the receiver sensitivity. The interaction between the probe (signal) photon and the target can be described by a beam splitter of transmissivity *T*^(*r*)^. Therefore, we can model the radar transmitter-target-radar receiver (directly reflected mode) channel (direct return channel) as a lossy thermal Bosonic channel

(3) $${\hat{a}}_{\mathrm{Rx}}^{\left(r\right)}\left(\varphi \right)=\sqrt{T^{\left(r\right)}}e^{-j\varphi^{\left(r\right)}}{\hat{a}}_{s}^{}+\sqrt{1-T^{\left(r\right)}}{\hat{a}}_{b}^{\left(r\right)},$$

where ${\hat{a}}_{b}^{\left(r\right)}$ is a background (thermal) state of the direct return channel with the mean photon number being $\left(1-T^{\left(r\right)}\right)\langle {\hat{a}}_{b}^{\left(r\right)†}{\hat{a}}_{b}^{\left(r\right)}\rangle =N_{b}$. With *φ*^(*r*)^ we denoted signal-mode phase shift introduced by the target and channel. The idler-mode channel is also modelled as the lossy and noisy Bosonic channel

(4) $${\hat{a}}_{\mathrm{Rx},\;\;\mathrm{idler}}^{}=\sqrt{T^{\left(i\right)}}{\hat{a}}_{i}^{}+\sqrt{1-T^{\left(i\right)}}{\hat{a}}_{b}^{\left(i\right)},$$

where *T*^(*i*)^ is transmissivity of the idler channel and ${\hat{a}}_{b}^{\left(i\right)}$ is the annihilation operator of the background (thermal) mode of the idler channel with the mean photon number being $\left(1-T^{\left(i\right)}\right)\langle {\hat{a}}_{b}^{\left(i\right)†}{\hat{a}}_{b}^{\left(i\right)}\rangle =N_{b}^{\left(i\right)}$. The radar returned probe and retained reference (stored idler) can be described by the following covariance matrix:

(5) $$\Sigma_{t}=\left[\begin{array}{cc} \left(2N_{s}+1\right)1 & 2\sqrt{T^{\left(r\right)}T^{\left(i\right)}N_{s}\left(N_{s}+1\right)}Z\delta_{1t}\\ 2\sqrt{T^{\left(r\right)}T^{\left(i\right)}N_{s}\left(N_{s}+1\right)}Z\delta_{1t} & \left(2N_{s}^{\left(r\right)}+1\right)1 \end{array}\right],$$

where $N_{s}^{\left(r\right)}=(T^{\left(i\right)}N_{s}+N_{b}^{\left(i\right)})T^{\left(r\right)}+N_{b}$. We use *t* to denote the target indicator. In the absence of the target, we have that *t* = 0 and in this case the return signal does not contain probe, just the background noise, and the covariance matrix is diagonal. On the other hand, in the presence of the target, we have that *t* = 1 and antidiagonal terms, representing the quantum correlation between the signal and idler, are non-zero.

**Figure 3:**
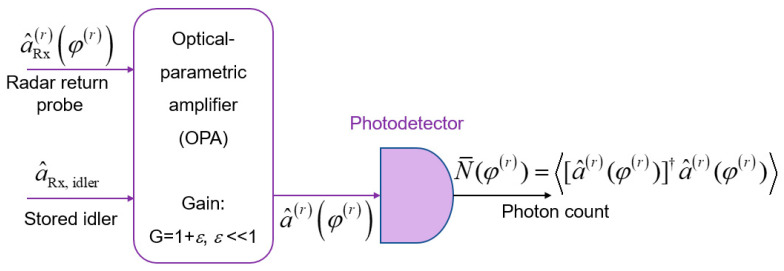
The optical-parametric amplifier (OPA)-based EA target detection receiver.

The EA monostatic radar receiver may use the optical parametric amplifier (OPA), shown in Figure 3, with a low gain *G* − 1 = *ε* << 1, to obtain:

(6) $${\hat{a}}^{\left(r\right)}\left(\varphi^{\left(r\right)}\right)=\sqrt{G}\;\;{\hat{a}}_{\mathrm{Rx},\;\;\mathrm{idler}}^{}+\sqrt{G-1}\;\;{\hat{a}}_{\mathrm{Rx}}^{\left(r\right)†}\left(\varphi^{\left(r\right)}\right)$$

for each signal-idler pair of a given mode. The direct detection of the OPA has the following mean photon number $\bar{N}\left(\varphi^{\left(r\right)}\right)=\langle {\left[{\hat{a}}^{\left(r\right)}(\varphi^{\left(r\right)})\right]}^{†}{\hat{a}}^{\left(r\right)}(\varphi^{\left(r\right)})\rangle$.

Zhang et al. have shown in ref. 19 that the OPA-based EA receiver, for ideal distribution of the idler (*T*^(*i*)^ = 1), provides ≤ 3 dB improvement over corresponding classical receiver. In the presence of experimental imperfections, the improvement was reduced to 1 dB, as shown in [19]. Given that the OPC receiver outperforms the OPA receiver [1,9,10], here we propose an EA joint monostatic-bistatic target detection scheme that employs the OPC on the transmitter side and classical coherent detection on both receiving ends, with details provided in following section.

## 3. Proposed Entanglement Assisted Joint Monostatic-Bistatic Radar Detection Scheme

**Figure 4:**
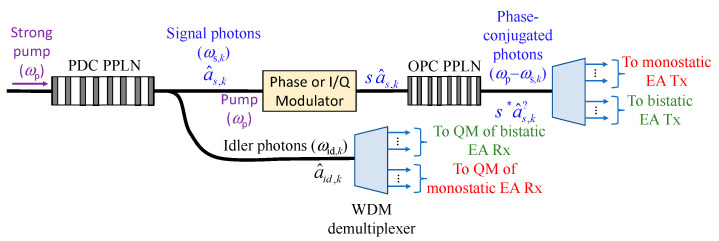
Joint monostatic-bistatic LiNbO_3_ technology-based integrated EA transmitter with transmit side OPC. PDC: parametric down conversion, OPC: optical phase-conjugation, PPLN: periodically poled LiNbO_3_ waveguide, QM: quantum memory.

In this section, we describe our proposed entanglement assisted joint monostatic-bistatic radar detection concept, shown in Figure 1, which is inspired by our recently proposed EA communication system [10]. The proposed joint monostatic-bistatic integrated (LiNbO_3_ technology-based) EA transmitter, with transmit side OPC, is provided in Figure 4. The phase modulator or I/Q modulator is optional here. We perform the OPC operation through the difference frequency generation (DFG) process by using the periodically poled LiNbO_3_ (PPLN) waveguide. In the first PPLN waveguide, the SPDC concept is utilized to generate signal-idler photon pairs, which get separated by the Y-junction. Given that the SPDC is the wideband process, a large number of signal-idler photon pairs are generated so that we use subscript *k* to denote the *k*th signal-*k*th idler photon pair. In the second PPLN, the DFG interaction of the pump photon *ω*_p_ and signal photon *ω*_s,*k*_ takes place and the phase-conjugated (PC) photon at radial frequency *ω*_p_−*ω*_s,*k*_ is generated. We further use the wavelength division demultiplexer to separate the signal/idler photons corresponding to monostatic and bistatic transmitters/receivers, as shown in Figure 1. As an illustrative example, for the strong pump at *λ*_p_ = 780 nm, through the SPDC the following signal-idler pairs can be generated: (1) the idler photon 1 at *λ*_i,1_ = 1535 nm—the signal photon 1 at wavelength *λ*_s,1_ = 1585.8 nm and (2) the idler photon 2 at *λ*_i,2_ = 1545 nm—the signal photon 2 at wavelength *λ*_s,2_ = 1575.3 nm. After the OPC PPLN waveguide, the signal photon 1 interacts with the pump photon through DFG to get the PC signal photon at *λ*_s,1,PC_ = 1/(1/*λ*_p_ − 1/*λ*_s,1_) = 1530 nm, which is the same wavelength as that of the idler photon 1. In a similar fashion, after the OPC PPLN waveguide the signal photon 2 interacts with the pump photon through DFG to get the PC signal photon at *λ*_s,2,PC_ = 1/(1/*λ*_p_ − 1/*λ*_s,2_) = 1545 nm, representing the same wavelength as that of the idler photon 2. In Figure 4 we use *s* to denote a signal constellation point imposed by either phase modulator or I/Q modulator. For M-ary PSK *s* is simply exp(j*θ*_mod_), where *θ*_mod_ ∈ {0,2π/*M*, …, (*M*−1) 2π/*M*}.

**Figure 5:**
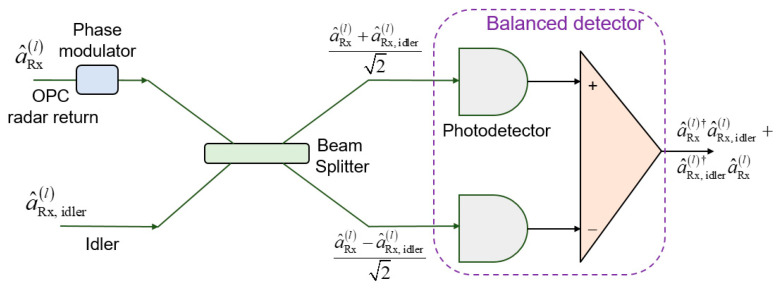
EA homodyne balanced detection receiver corresponding to the direct return/forward scattered components. The phase modulator is used to detect either in-phase or quadrature component of the OPC signal. Photodiode responsivity is set to 1 A/W.

By performing the OPC on the transmitter side, conventional-classical balanced coherent detection receiver can be applied on receive sides of monostatic and bistatic radars (see Figure 1), with one such receiver being provided in Figure 5. Evidently, the OPC radar direct return probe/forward scattered probe and idlers modes are mixed on balanced beam splitter, followed by two photodiodes. The idler mode for each EA detector serves as a local (oscillator) laser signal for the homodyne coherent detection.

For transmit-side OPC, the direct return channel *r*/forward scattering channel *fs* models can be represented by

(7) $${\hat{a}}_{Rx,k}^{\left(l\right)}\left(\varphi^{\left(l\right)}\right)=\sqrt{T^{\left(l\right)}}e^{-j\varphi^{\left(l\right)}}{\hat{a}}_{s,k}^{\left(l\right)†}+\sqrt{1-T^{\left(l\right)}}{\hat{a}}_{b}^{\left(l\right)},$$

where in the superscript *l* is used to denote either the direct return channel (*l* = *r*) or the forward scattering channel (*l* = *fs*), while subscript *k* is used to denote the *k*th signal-idler photon pair. The overall phase *φ*^(*l*)^ is composed of three components:

(8) $$\varphi^{\left(l\right)}=\theta_{\mathrm{mod}}+\vartheta^{\left(l\right)}+\phi^{\left(l\right)},$$

where *θ*_mod_ is the modulation phase (when M-ary PSK is used), while $\vartheta^{\left(l\right)}$ denotes the phase-shift introduced by the target. For the direct return probe, given that the distance between the transceiver and target is *d*, the phase shift introduced by the target will be $\vartheta^{\left(r\right)}=2kd$, with *k* being the wave number related to the wavelength λ by *k* = 2π/λ. On the other hand, given that the distance between target and receiver in the forward scattering channel is *D*, the corresponding phase shift introduced by the target will be $\vartheta^{\left(fs\right)}=k(d+D)$. Finally, *φ*^(*l*)^ is the random phase shift introduced by the *l*th channel. The purpose of the transmit side phase modulator is to impose the sequence on the transmitter side that will be used for estimation of the random phase shift and corresponding cancelation.

The balanced detector (BD) photocurrent operator (assuming that the photodiode responsivity is 1 A/W) for EA detector, shown in Figure 5, is given by:

(9) $${\hat{i}}_{BD}^{\left(l\right)}={\left({\hat{a}}_{Rx}^{\left(l\right)}\right)}^{†}{\hat{a}}_{\mathrm{Rx},\mathrm{idler}}^{\left(l\right)}+{\left({\hat{a}}_{\mathrm{Rx},\mathrm{idler}}^{\left(l\right)}\right)}^{†}{\hat{a}}_{Rx}^{\left(l\right)},\;\;l\in \left\{r,fs\right\}$$

For the receive side phase modulator shift of ∆*φ* = 0 rad (see Figure 5), in the presence of the target, we obtain the following BD photocurrent operator expectation:

(10) $$\langle {\hat{i}}_{BD}^{\left(l\right)}\rangle =2\sqrt{T^{\left(i\right)}T^{\left(l\right)}N_{s}\left(N_{s}+1\right)}\cos\varphi^{\left(l\right)},\;\;l\in \left\{r,\;\;fs\right\}$$

On the other hand, for the receive side phase modulator shift of ∆*φ* = −π/2 rad, in the presence of target, we obtain the following BD photocurrent operator expectation:

(11) $$\langle {\hat{i}}_{BD}^{\left(l\right)}\rangle =2\sqrt{T^{\left(i\right)}T^{\left(l\right)}N_{s}\left(N_{s}+1\right)}\sin\varphi^{\left(l\right)},\;\;l\in \left\{r,\;\;fs\right\}$$

In order to determine the exact phase-shift and the target range both in-phase and quadrature components are needed.

For the receive side phase modulator shift of ∆φ = 0 rad, the variance of the BD photocurrent operator, defined as $\mathrm{Var}\left({\hat{i}}_{BD}^{\left(l\right)}\right)=\langle {\left({\hat{i}}_{BD}^{\left(l\right)}\right)}^{2}\rangle -{\langle {\hat{i}}_{BD}^{\left(l\right)}\rangle }^{2}$, will be:

(12) $$\mathrm{Var}\left({\hat{i}}_{BD}^{\left(l\right)}\right)=N_{i}N_{s}^{\left(l\right)}+\left(N_{i}+1\right)\left(N_{s}^{\left(l\right)}+1\right)\;+2N_{s}T^{\left(l\right)}T^{\left(i\right)}(N_{s}+1)\left[\cos\left(2\varphi^{\left(l\right)}\right)-2\cos^{2}\varphi^{\left(l\right)}\right],$$

where $N_{s}^{\left(l\right)}=(T^{\left(i\right)}N_{s}+N_{b}^{\left(i\right)})T^{\left(l\right)}+N_{b}^{\left(l\right)}$.

In the absence of the target, the BD photocurrent operator expectation is zero, while the corresponding variance is:

(13) $$\mathrm{Var}\left({\hat{i}}_{BD,t=0}^{\left(l\right)}\right)=N_{i}N_{b}^{\left(l\right)}+\left(N_{i}+1\right)\left(N_{b}^{\left(l\right)}+1\right)\;=N_{s}N_{b}^{\left(l\right)}+\left(N_{s}+1\right)\left(N_{b}^{\left(l\right)}+1\right),$$

where we used the fact that *N*_i_ = *N*_s_.

Given that in the target detection problem the prior probabilities are not known in advance we need to apply the Neyman-Pearson criterion [20,21]. In Neyman-Pearson criterion we fix the maximum tolerable false alarm probability and maximize the target detection probability.

For the proposed EA joint monostatic-bistatic target detection scheme, the *false alarm (FA) probability* is given by:

(14) $$Q_{FA}=\frac{1}{2}\mathrm{erfc}\left(\frac{t_{sh}}{\sqrt{N_{s}N_{b}+(N_{s}+1)(N_{b}+1)}}\right),$$

where *t_sh_* is the threshold determined from the tolerable FA probability, wherein the complementary error function is given by $\mathrm{erfc}(x)=(2/\sqrt{\pi })\int_{x}^{\infty }\exp(-u^{2})du$.

Assuming that the equal gain combining (see ref. [22] for more details) is used as the joint detection scheme for two receivers, the target *detection probability* is given by:

(15) $$Q_{D}=\frac{1}{2}\mathrm{erfc}\left(\frac{t_{sh}-m_{v}}{\sqrt{V^{\left(r\right)}+V^{\left(fs\right)}}}\right),$$

where

(16) $$\begin{array}{l} m_{v}=2\sqrt{T^{\left(r\right)}T^{\left(i\right)}N_{s}(N_{s}+1)}+2\sqrt{T^{\left(fs\right)}T^{\left(i\right)}N_{s}(N_{s}+1)},\\ V^{\left(r\right)}=N_{i}N_{s}^{\left(r\right)}+\left(N_{i}+1\right)\left(N_{s}^{\left(r\right)}+1\right)\;-2T^{\left(r\right)}T^{\left(i\right)}N_{s}(N_{s}+1),\\ V^{\left(fs\right)}=N_{i}N_{s}^{\left(fs\right)}+\left(N_{i}+1\right)\left(N_{s}^{\left(fs\right)}+1\right)\;-2T^{\left(fs\right)}T^{\left(i\right)}N_{s}(N_{s}+1). \end{array}$$

## 4. Illustrative Numerical Results

The referent case will be the monostatic radar in which a coherent state is used to illuminate the target, in the presence of thermal (background) radiation. The density operator, in the presence of thermal radiation, has the following P-representation [1,2,3,4,20]:

(17) $$\rho_{t}=\frac{1}{\pi N_{b}}\int e^{-\frac{{\left|\alpha -\mu_{t}\right|}^{2}}{N_{b}}}|\alpha \rangle \langle \alpha |d^{2}\alpha .$$

In the absence of the target (*t* = 0) we have that *μ*_0_ = 0, while in the presence of the target (*t* = 1) *μ*_1_ = *μ*. The parameter *N*_b_ denotes the average number of thermal (background) photons. The coherent state |α〉 can be expressed in terms of number states by $|\alpha \rangle =e^{-{\left|\alpha \right|}^{2}/2}\sum_{n}(\alpha^{n}/\sqrt{n!})|n\rangle$ and after substitution in (17) we obtain:

(18) $$\rho_{0}=\sum_{n=0}^{\infty }\left(1-v\right)v_{}^{n}|n\rangle \langle n|,\;\;\;v=N_{b}/(N_{b}+1).$$

The corresponding density matrix in the presence of target is given by (20):

(19) $$\langle n|\rho_{1}|m\rangle =\left\{\begin{array}{c} \begin{array}{l} \left(1-v\right)\sqrt{\frac{n!}{m!}}v_{}^{m}{\left(\mu_{}^{*}/N_{}\right)}^{m-n}e^{-\left(1-v\right){\left|\mu \right|}^{2}}\cdot \\ L_{n}^{m-n}\left[-{\left(1-v\right)}^{2}{\left|\mu \right|}^{2}/v\right],\;\;m\geq n \end{array}\\ {\langle m|\rho_{k}|n\rangle }^{*},\;\;m<n \end{array}\right.$$

where |*μ*〉 denotes the state used to illuminate the target. In (19), we use $L_{deg}^{ord}(\cdot )$ to denote the associated Laguerre polynomials with superscript *ord* and subscript *deg* denoting the order and degree, respectively. For the Neyman-Pearson criterion the optimum strategy will be to determine the eigenvalues *η_k_* and eigenkets $|\eta_{k}\rangle$ of the operator $\rho_{1}-\Lambda \rho_{0}$ by solving the eigenvalue equation:

(20) $$\left(\rho_{1}-\Lambda \rho_{0}\right)|\eta_{k}\rangle =\eta_{k}|\eta_{k}\rangle ,$$

in which the parameter Λ is determined from the maximum tolerable FA probability. This problem can be solved numerically.

To reduce receiver complexity, the *Helstrom threshold detector* can be used instead (20), with the corresponding detection operator defined as

(21) $$\Pi_{H.t.}^{}={\left(N_{b}+0.5\right)}^{-1}\left(\hat{a}+{\hat{a}}^{†}\right),$$

which is related to the in-phase operator.

**Figure 6:**
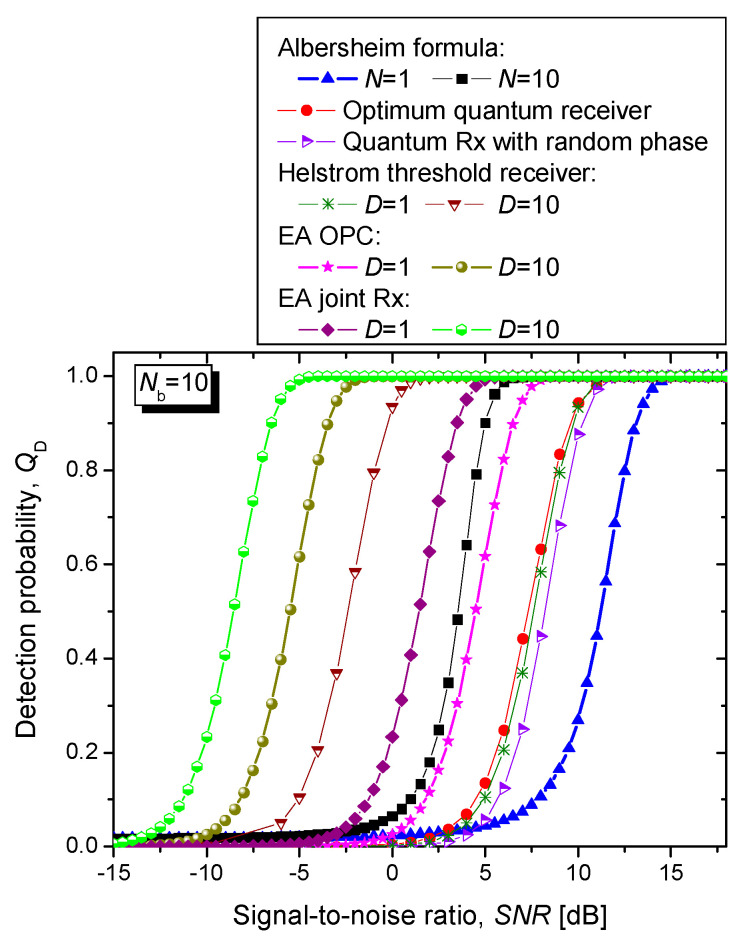
Detection probability vs. SNR [dB] for different radar detection schemes for average number of thermal photons set to *N_b_* = 10. The maximum tolerable FA probability is fixed to *Q*_FA_ = 10^−6^. The monostatic and bistatic idler channels are assumed to be ideal.

By assuming that the idler channels are ideal by setting the corresponding transmissivities to *T*^(*l*)^ = 1, in Figure 6 we compare the proposed EA joint monostatic-bistatic target detection scheme against various coherent states-based schemes and EA detection scheme for monostatic radar, in terms of detection probability vs. SNR, by setting the average number of background photons to *N_b_* = 10, wherein the false alarm probability that can be tolerated is fixed to *Q*_FA_ = 10^−6^. For completeness of the presentation, the classical Albersheim’s equation-based curves are provided as well for the number of samples set to *N* = 1 and 10 (see [23,24] for the Albersheim’s equation details). For the non-classical target detection schemes the SNR is defined by *N_s_*/(2*N_b_* + 1). The coherent states-based detection schemes under study include optimum quantum detector, quantum receiver (Rx) with the random phase, and Helstrom threshold receiver. Evidently, the proposed EA joint (monostatic-bistatic) target detection scheme significantly outperforms various coherent states-based detections schemes, the EA detection scheme for monostatic radar, and the classical target detection.

Given that the SPDC-based entangled source is broadband source in Figure 6 we also study the improvement in SNR that we can get when the number of bosonic modes is increased to *D* = 10. The proposed EA joint target detection scheme significantly outperforms the Helstrom threshold receiver with *D* = 10 modes and classical radar detector for *N* = 10 samples. For the detection probability set to *Q*_D_ = 0.95 (and false alarm probability fixed to *Q*_FA_ = 10^−6^), the EA target detection scheme for *D* = 10 Bosonic modes outperforms the Helstrom detection scheme (for the same number of Bosonic modes) by 6.16 dB, while at the same time outperforming the corresponding classical scheme with *N* = 10 samples by even 11.29 dB. The joint EA scheme for *D* = 10 bosonic modes outperforms the corresponding EA scheme for monostatic radar (also with 10 bosonic modes at *Q*_D_ = 0.95) by 3.01 dB.

**Figure 7:**
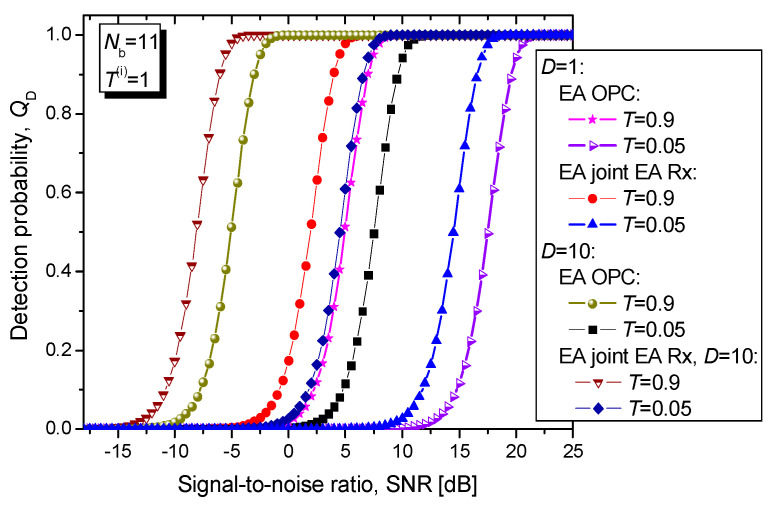
Detection probability vs. SNR [dB] for joint EA scheme for different direct return probe/forward scattered probe bosonic channel transmissivities *T*^(*r*)^ = *T*^(*fs*)^ = *T*. The maximum tolerable false alarm probability is fixed to *Q*_FA_ = 10^−6^. The idler channel is assumed to be ideal.

In Figure 7 we evaluate the proposed EA joint detection scheme’s detection probability vs. SNR by modelling both the direct return probe and forward scattered probe channels as the bosonic noisy and lossy channels with *N*_b_ = 11 and transmissivities *T*^(*r*)^ = *T*^(*fs*)^ = *T*, wherein the corresponding channel models are given by Equation (7). Here we assume the ideal distribution of entanglement over the idler channels (*T*^(*i*)^ = 1 and *N_b_*^(*i*)^ = 0). Clearly, when transmissivities of the direct return probe and forward scattered probe channels are low, the use of single Bosonic mode is not sufficient because the required SNR to achieve high target detection probability is way too high. On the other hand, when the number of bosonic modes is increased to 10, high target detection probabilities can be achieved even at moderate SNRs (for low channel transmissivities). For *T* = 0.05, the EA joint detector with 10 bosonic modes outperforms EA monostatic radar detector by 3.04 dB at *Q*_D_ = 0.95.

**Figure 8:**
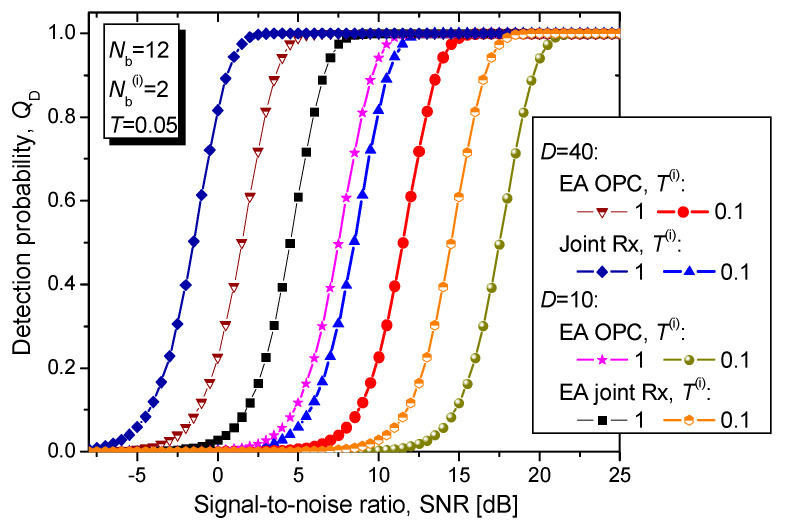
Detection probability vs. SNR [dB] for EA joint detection scheme for different idler channels transmissivities. The direct return probe/forward scattered probe bosonic channel transmissivities are fixed to *T*^(*r*)^ = *T*^(*fs*)^ = *T* = 0.05. The maximum tolerable false alarm probability is set to *Q*_FA_ = 10^−6^.

In Figure 8 we evaluate the proposed EA joint detection scheme’s detection probability vs. SNR by fixing the direct return probe/forward scattered probe channel transmissivities to *T^(r)^* = *T^(fs)^* = *T* = 0.05 and varying the transmissivity of the idler channels, wherein the idler channel model is described by Equation (4). Both signal and idler bosonic channels are under assumption of being noisy with corresponding parameters being *N_b_* = 12 and *N_b_*^(*i*)^ = 2, respectively. Obviously, when the idler channel is noisy and lossy the same detection probability is achieved for higher SNR values, compared to the case with perfect distribution of entanglement. To solve for this problem, we can increase the number of bosonic modes, which is not difficult to implement thanks to the wideband nature of the SPDC process.

**Figure 9:**
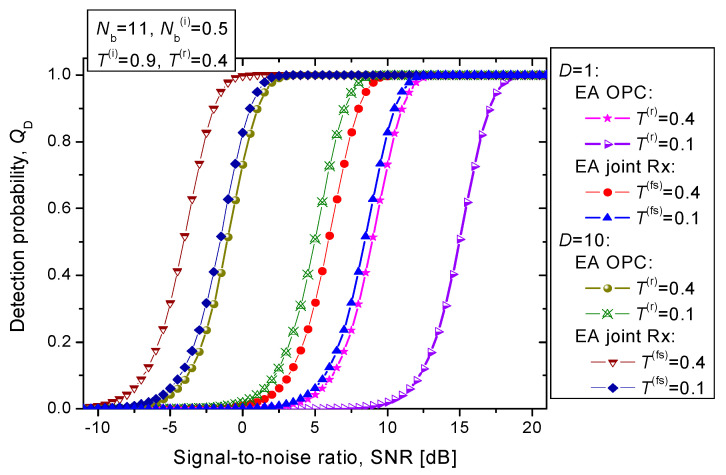
Detection probability vs. SNR [dB] for EA joint detection scheme for fixed idler channels transmissivity *T*^(*i*)^ = 0.9. The direct return probe channel transmissivity is set to *T*^(*r*)^ = 0.4, while the forward scattered probe channel transmissivity is varied *T*^(*fs*)^ ∈ {0.1, 0.4}. The maximum tolerable false alarm probability is fixed to *Q*_FA_ = 10^−6^.

Finally, in Figure 9 we study the proposed EA joint detection scheme’s detection probability when the transmissivities of the direct return probe and the forward scattered probe channels are different, while the average number of thermal photons is set to *N*_b_ = 11. The idler channels are considered identical but lossy and noisy [*T*^(i)^ = 0.9 and *N*_b_^(i)^ = 0.5]. The joint EA detection scheme for *T*^(*r*)^ = 0.4 and *T*^(*fs*)^ = 0.1 for 10 bosonic modes outperforms the EA detector for monostatic radar with *T*^(*r*)^ = 0.4 by even 6.49 dB at *Q*_D_ = 0.95.

## 5. Concluding Remarks

We have proposed the entanglement assisted joint bistatic-monostatic quantum radar detection scheme. The proposed EA joint radar detection scheme employs the optical phase conjugation on the transmitter side and classical coherent detection on both receiver sides.

The proposed EA joint target detection scheme has been evaluated against the coherent states-based quantum detection schemes and EA detection scheme for monostatic radar. We have shown that the detection probability of the proposed EA joint target detection scheme has been significantly better than that of corresponding coherent states-based quantum detection schemes, the classical detection, and EA detection scheme for monostatic radar. The proposed scheme has been also evaluated by assuming the imperfect distribution of entanglement and by modeling the direct return probe and forward scattered probe channels as both lossy and noisy Bosonic channels. The proposed EA joint transmitter, with transmit side OPC, is suitable for implementation in mature LiNbO_3_ technology. Given that the EA receiver is based on a commercially available balanced coherent detector, the implementation of the proposed joint bistatic-monostatic radar is not far from practical implementation.

## Data Availability

Not applicable.
